# What If Leisure Time Activities Were a Solution for Athletes' Long-Term Development and Health?

**DOI:** 10.3389/fpsyg.2022.926419

**Published:** 2022-07-06

**Authors:** Philippe Vacher, Nadia Sondt, Guillaume Levillain, Cyril Bossard, Magali Prost, Marjorie Bernier

**Affiliations:** Research Center for Education Learning and Didactics (EA 3875), University of West Brittany, Brest, France

**Keywords:** adolescent athletes, leisure, performance, recovery, sustainable practice

## Introduction

Adolescents who wish to pursue a career as a high-performance athlete are expected to be engaged in a sport specialization process. In this way, they have to adopt an intense and specific focus on one sport and exclude others. Although youth sports participation has been linked to numerous physical, psychological and social benefits, it has also been associated with negative outcomes such as low self-esteem, aggressive behaviors or eating disorders (Fraser-Thomas et al., 2005). The modern integrated approach underlines that the youth athletes system is organized around three main subsystems (i.e., family, team and environmental subsystems), which are interrelated and impact athletes' development and practice (Dorsch et al., 2022).

Then, in addition to the constraints and goals of the sport, the life and career of modern adolescent athletes are characterized, in many countries, by the requirement to undertake education alongside their sports training. Studies that have investigated dual career (DC) in student-athletes have shown that this population experiences high demands across athletic and non-athletic levels of development (Brown et al., 2015). These studies also highlighted that student-athletes have to balance their time and energy to fulfill their commitments in different life areas, bringing out the time management challenge between time constraints and leisure time, organizational capacity and long-term development (Stambulova et al., 2021).

Sport and educational commitments are typically associated with entrance into academies or clubs with high prestige. In order to facilitate athletes' participation and achievement in education and sports programs, many countries set up Sports and Academic Training Centers (SATCs). SATCs aim to offer athletes all the resources they need from an academic and sports point of view. However, despite the SATCs support, it has been recently highlighted that the sports specialization process still predisposes adolescent athletes to social isolation, poor academic performance, emotional disturbances, intense and chronic stress states, lack of proactive recovery strategies, decreased family time, and burnout (Brenner et al., 2019). In response to these health concerns and behaviors, several health organizations have proposed guidelines and position statements to guide parents and practitioners toward best practices for managing young athletes. A recent literature review (Herman et al., 2022) highlighted that the most common recommendations endorsed the following concepts: “monitor athlete wellbeing,” “youth athletes need access to well trained, quality coaches,” “multi-sport participation,” “limit early organized participation and/or training,” and “parents require awareness of training, coaching, and best practices.” However, Herman et al.'s study (Herman et al., 2022) highlighted that: (a) the level of evidence provided to support a given recommendation varied significantly; (b) the level of detail and the consistency of terms used throughout the results were typically low, and (c) recommendations were frequently made without reference to potential outcome measures or specific strategies that could be used for practical implementation in the community.

According to the recent literature, there is a lack of applied strategies that may help student-athletes to cope with the various stressors and constraints related to the sport specialization period of their careers, that could lead to dysfunctional states. Beyond the positive and negative aspects of early sport specialization for long term development, performance, health and sport participation (LaPrade et al., 2016), we argued that it would be a fundamental perspective in sports science to validate interventional protocols in this way. Considering the specificity of adolescent student-athletes that are engaged in sport specialization, it appears that these athletes are exposed to the increased sport and educational constraints in their lifestyle, leading to time pressure. This time pressure is expressed as a lack of time for physical, psychological and own needs recovery, which is a psychological condition and burnout significant risk factor (Sorkkila et al., 2020). In this line, we assume that adolescence is a pivotal period for athletes' development because most mental health disorders occur in late adolescence and early adulthood, as well as identity formation and individuation (Sutcliffe and Greenberger, 2020). Especially, adolescent athletes that have to commit in sport specialization are an at-risk population in the way that they may develop a unique identity (i.e., athlete identity), which is risky when things go wrong as an athlete (injuries, setbacks, etc.), leading adolescents' foundation to be shaken (Champ et al., 2020). Therefore, investigating new strategies that consider the problem of time pressure and the need for leisure activities to ensure adolescents' functional development and maturation is of crucial interest for a long-term performance perspective and a sustainable sports practice.

## Leisure Activities Approach

While physical and mental recovery in sport have received increasing attention in research and practice the past decades (Kellmann, 2010), we notice that leisure activities is underexamined in sports science, perhaps because of the culture of effort, work and surpassing oneself attached to sport performance.

Leisure is defined as how one's spends their free time, and is considered as being the “*principal driving force underpinning the human desire to render life meaningful… or to give it the sense of passion, pleasure and purpose*” (Blackshaw, 2017). These voluntary non-work activities (Hills and Argyle, 1998) are engaged in for enjoyment and encompass actions such as: taking part in hobbies; participating in arts; taking educational classes; reading; socializing; shopping; listening to music; engaging in libraries or culture; cooperating in community, neighborhood, or tenants' groups; and participating in social clubs (Fancourt et al., 2021). Accordingly, thousands of studies have shown a relationship between leisure engagement and both physical and mental health (e.g., mental health: prevention and management of mental illnesses such as depression, anxiety, stress; physical health: chronic pain, frailty, coronary heart disease and disability) (see Hills and Argyle, 1998 for a review).

According to the Multi-level Leisure Mechanisms Framework (Fancourt et al., 2021), leisure activities are complex interventions in that they contain individual and group level components and outcomes (Craig et al., 2013). To date, over 600 potential mechanisms of action by which leisure activities might affect health have been identified and categorized into psychological, biological, social and behavioral processes (Fancourt et al., 2021). Some of these mechanisms operate at the micro-level (i.e., affect individuals or tiny groups). In contrast, others operate at the meso-level (i.e., affect larger groups, communities, and institutions) or at the macro-level (i.e., affect societies and cultures at large). To quickly summarize the literature, some of these mechanisms can be directly activated by leisure management and engagement (and will have an immediate effect on health), while others are part of more complex indirect pathways and will affect health from a long-term perspective (Fancourt et al., 2021). Then, considering leisure activities requests to keep in mind that (a) no leisure activity will activate just one causal mechanism (Rogers, 2008); (b) leisure activities effects are the result of the interaction of multiple different mechanisms (Preiser et al., 2018); leisure activities interventions mechanisms are non-linear and involve feedback loops and recursive causality (Rogers, 2008; Preiser et al., 2018). Finally, leisure mechanisms only make sense when they are considered from a dynamic systems perspective (Kernick, 2006), rather than on static states and protocols (Fancourt et al., 2021).

## Discussion

Despite the central role of leisure activities for individual development and health, we failed to find studies in sports science that investigates applied protocols in athletes. In the general population, organized leisure-time activities (OLTA) have been identified as allowing the individual strengths of adolescents to be aligned with developmental assets (Bowers et al., 2014). OLTA represents a wide range of activities taking place during leisure time outside the regular school curriculum (Bohnert et al., 2010). These activities can be characterized as having a structure with defined rules and goals, being supervised by adults, having a regular schedule and putting emphasis on skill-building (Larson, 2000; Mahoney et al., 2005). Literature has shown associations between OLTA and relevant healthy youth development factors in adolescents (Bohnert et al., 2010; Farb and Matjasko, 2012). OLTA recognized benefits in the general population cover protections against risk behaviors, reduction in substance abuse consumption, delinquency, or bullying of others (Farb and Matjasko, 2012). It has also been shown that OLTA may be a way to channel some stress-reduction efforts (Darling, 2005) and negate the need for stabilization of one's social position through risk behaviors (Viau and Poulin, 2015; Viau et al., 2015), giving social support for identity-related exploration. Finally, studies have shown that one's participation in OLTA leads to an increase in his/her experience to higher levels of intrinsic motivation and challenge at the same time (Hansen et al., 2010), promoting the development of initiative, identity formation, and the building of teamwork skills and social capital (Hansen et al., 2003).

All of this seems to link OLTA to healthy developmental outcomes (Farb and Matjasko, 2012), which may be of key interest for adolescent student-athletes. This suggests that specific leisure mechanisms and applied protocols should be investigated in student-athletes, mainly based on longitudinal protocols and ecological conditions. For example, it would be interesting to investigate how applied protocols based on OLTA recommendations could help adolescent athletes preserve their recovery-stress states around real training seasons. An important challenge for these protocols will be to carefully manage how “adolescents” activities will be organized. Indeed, it has been shown that adolescents' participation in organized activities has protective effects while overscheduling induces major concerns in this population (Badura et al., 2015). In this line, a quantitative and qualitative approach should be relevant in order to individualize interventions and to take into account the key 'athletes' biopsychosocial aspects in a systemic approach. This research field may benefit from the extensive literature that exists on the general and disease population. It may help develop specific models and applied strategies to ensure a long-term sustainable practice and psychological/physical/social development of adolescent athletes.

## Author Contributions

PV, NS, GL, CB, MP, and MB were involved in the manuscript preparation. All authors contributed to the article and approved the submitted version.

## Conflict of Interest

The authors declare that the research was conducted in the absence of any commercial or financial relationships that could be construed as a potential conflict of interest.

## Publisher's Note

All claims expressed in this article are solely those of the authors and do not necessarily represent those of their affiliated organizations, or those of the publisher, the editors and the reviewers. Any product that may be evaluated in this article, or claim that may be made by its manufacturer, is not guaranteed or endorsed by the publisher.

## References

[B1] Badura P. Geckova A. M. Sigmundova D. Van Dijk J. P. Reijneveld S. A. (2015). When children play, they feel better: organized activity participation and health in adolescents energy balance-related behaviors. BMC Public Health 15, 1–8. 10.1186/s12889-015-2427-5 26499458 PMC4619483

[B2] Blackshaw T. (2017). Re-Imagining Leisure Studies. London: Routledge, 184.p.

[B3] Bohnert A. Fredricks J. Randall E. (2010). Capturing unique dimensions of youth organized activity involvement: theoretical and methodological considerations. Rev. Educ. Res. 80, 576–610. 10.3102/0034654310364533

[B4] Bowers E. P. John Geldhof G. Johnson S. K. Lerner J. V. Lerner R. M. (2014). Special issue introduction: thriving across the adolescent years: a view of the issues. J. Youth Adolesc. 43, 859–868. 10.1007/s10964-014-0117-8 24723047

[B5] Brenner J. S. LaBotz M. Sugimoto D. Stracciolini A. (2019). The psychosocial implications of sport specialization in pediatric athletes. J. Athl. Train. 54, 1021–1029. 10.4085/1062-6050-394-18 31532693 PMC6805069

[B6] Brown D. J. Fletcher D. Henry I. Borrie A. Emmett J. Buzza A.. (2015). A British university case study of the transitional experiences of student-athletes. Psychol. Sport Exerc. 21, 78–90. 10.1016/j.psychsport.2015.04.002

[B7] Champ F. M. Ronkainen N. J. Littlewood M. A. Eubank M. (2020). Supporting identity development in talented youth athletes: insights from existential and cultural psychological approaches. J. Sport Psychol. Action 11, 219–232. 10.1080/21520704.2020.1825027

[B8] Craig P. Dieppe P. Macintyre S. Michie S. Nazareth I. Petticrew M.. (2013). Developing and evaluating complex interventions: the new medical research council guidance. Int. J. Nurs. Stud. 50, 587–592. 10.1016/j.ijnurstu.2012.09.010 23159157

[B9] Darling N. (2005). Participation in extracurricular activities and adolescent adjustment: cross-sectional and longitudinal findings. J. Youth Adolesc. 34, 493–505 10.1007/s10964-005-7266-816802902

[B10] Dorsch T. E. Smith A. L. Blazo J. A. Coakley J. Côté J. Wagstaff C. R. D.. (2022). Toward an integrated understanding of the youth sport system. Res. Q. Exerc. Sport. 93, 105–119. 10.1080/02701367.2020.1810847 32960153

[B11] Fancourt D. Aughterson H. Finn S. Walker E. Steptoe A. (2021). How leisure activities affect health: a narrative review and multi-level theoretical framework of mechanisms of action. Lancet Psychiatry 8, 329–339. 10.1016/S2215-0366(20)30384-9 33581775 PMC7613155

[B12] Farb A. F. Matjasko J. L. (2012). Recent advances in research on school-based extracurricular activities and adolescent development. Dev. Rev. 32, 1–48. 10.1016/j.dr.2011.10.001

[B13] Fraser-Thomas J. L. Côté J. Deakin J. (2005). Youth sport programs: an avenue to foster positive youth development. Phys. Educ. Sport Pedagog. 10, 19–40. 10.1080/1740898042000334890

[B14] Hansen D. M. Larson R. W. Dworkin J. B. (2003). What adolescents learn in organized youth activities: a survey of self-reported developmental experiences. J. Res. Adolesc. 13, 25–55. 10.1111/1532-7795.1301006

[B15] Hansen D. M. Skorupski W. P. Arrington T. L. (2010). Differences in developmental experiences for commonly used categories of organized youth activities. J. Appl. Dev. Psychol. 31, 413–421. 10.1016/j.appdev.2010.07.001

[B16] Herman D. C. Nelson V. R. Montalvo A. M. Myer G. D. Brenner J. S. DiFiori J. P.. (2022). Systematic review of health organization guidelines following the AMSSM 2019 youth early sport specialization summit. Sport Heal. A Multidiscip. Approach 14, 127–134. 10.1177/19417381211051371 34668459 PMC8669928

[B17] Hills P. Argyle M. (1998). Positive moods derived from leisure and their relationship to happiness and personality. Pers. Individ. Dif. 25, 523–535. 10.1016/S0191-8869(98)00082-8

[B18] Kellmann M. (2010). Preventing overtraining in athletes in high-intensity sports and stress/recovery monitoring. Scand. J. Med. Sci. Sports 20, 95–102. 10.1111/j.1600-0838.2010.01192.x 20840567

[B19] Kernick D. (2006). Wanted–new methodologies for health service research. Is complexity theory the answer? Fam. Pract. 23, 385–390. 10.1093/fampra/cml011 16597669

[B20] LaPrade R. F. Agel J. Baker J. Brenner J. S. Cordasco F. A. Côté J.. (2016). AOSSM early sport specialization consensus statement. Orthop. J. Sport Med. 4, 232596711664424. 10.1177/2325967116644241 27169132 PMC4853833

[B21] Larson R. W. (2000). Toward a psychology of positive youth development. Am. Psychol. 55, 170–183. 10.1037/0003-066X.55.1.170 11392861

[B22] Mahoney J. L. Larson R. W. Eccles J. S. Lord H. (2005). “Organized activities as developmental contexts for children and adolescents,” in Organized Activities As Contexts of Development: Extracurricular Activities, After School and Community Programs.

[B23] Preiser R. Biggs R. De Vos A. Folke C. (2018). Social-ecological systems as complex adaptive systems: organizing principles for advancing research methods and approaches. Ecol. Soc. 23, art46. 10.5751/ES-10558-230446

[B24] Rogers P. J. (2008). Using programme theory to evaluate complicated and complex aspects of interventions. Evaluation 14, 29–48. 10.1177/1356389007084674

[B25] Sorkkila M. Ryba T. V. Selänne H. Aunola K. (2020). Development of school and sport burnout in adolescent student-athletes: a longitudinal mixed-methods study. J. Res. Adolesc. 30, 115–133. 10.1111/jora.12453 30207416

[B26] Stambulova N. B. Ryba T. V. Henriksen K. (2021). Career development and transitions of athletes: the international society of sport psychology position stand revisited. Int. J. Sport Exerc. Psychol. 19, 524–550. 10.1080/1612197X.2020.1737836

[B27] Sutcliffe J. H. Greenberger P. A. (2020). Identifying psychological difficulties in college athletes. J. Allergy Clin. Immunol. Pract. 8, 2216–2219. 10.1016/j.jaip.2020.03.006 32209401

[B28] Viau A. Denault A.-S. Poulin F. (2015). Organized activities during high school and adjustment one year post high school: identifying social mediators. J. Youth Adolesc. 44, 1638–1651. 10.1007/s10964-014-0225-5 25404238

[B29] Viau A. Poulin F. (2015). 'Youths' organized activities and adjustment in emerging adulthood: a multidimensional conception of participation. J. Res. Adolesc. 25, 652–667. 10.1111/jora.12159

